# Scaling up multispectral color filters with binary lithography and reflow (BLR)

**DOI:** 10.1515/nanoph-2024-0090

**Published:** 2024-06-03

**Authors:** Md Abdur Rahman, Soroosh Daqiqeh Rezaei, Deepshikha Arora, Hao Wang, Tomohiro Mori, Ser Chern Chia, John You En Chan, Parvathi Nair Suseela Nair, Siam Uddin, Cheng-Feng Pan, Wang Zhang, Hongtao Wang, Zheng Ruitao, Lim Sin Heng, Joel K. W. Yang

**Affiliations:** Engineering Product Development Pillar, 338939 Singapore University of Technology and Design, 8 Somapah Road, Singapore 487372, Singapore; 88299 Industrial Technology Center of Wakayama Prefecture, Wakayama 6496261, Japan; Institute of Materials Research and Engineering, A*STAR (Agency for Science, Technology and Research), 2 Fusionopolis Way, #08-03 Innovis, Singapore 138634, Singapore; Lite-On Singapore Pte. Ltd., New Tech Park, Singapore 556741, Singapore

**Keywords:** multispectral filters, structural colors, electron beam lithography, binary lithography and reflow

## Abstract

Efforts to increase the number of filters are driven by the demand for miniaturized spectrometers and multispectral imaging. However, processes that rely on sequential fabrication of each filter are cost ineffective. Herein, we introduce an approach to produce at least 16 distinct filters based on a single low-resolution lithographic step with minimum feature size of 0.6 μm. Distinct from grayscale lithography, we employ standard binary lithography but achieve height variations in polymeric resist through a post-development reflow process. The resulting transparent polymeric films were incorporated in Fabry–Perot cavity structures with cavity thickness ranging from 90 to 230 nm to produce transmittance across the visible spectrum. This binary lithography and reflow (BLR) process demonstrates control of the dielectric layer thickness down to ∼15 nm. This new process provides a cost-effective alternative to traditional techniques in fabricating microscopic transmission filters, and other applications where precise thickness variation across the substrate is required.

## 1 Introduction

Spectral sensors are an integral part in digital cameras and mobile phones. To exhibit color sensitivity, color filters are fabricated above photodiodes. These color filters are typically composed of organic dye filters [1]. Due to the lack of durability in high temperature, high-cost fabrication process and highly impractical for multispectral imaging where more than three primary colors are required, these are not a  highly feasible process to fabricate. There are several structures that can replace organic dye filters, such as diffraction gratings [2], plasmonic structures [3], [4], [5], [6], [7], [8], [9], [10], [11], structures with thin lossy-dielectrics coatings [12], dielectric metasurfaces with high refractive index [13], [14]. Nanostructuring of Si itself has been proposed to combine color filter and photodiode in a single element [15]. Though these structures do not require multiple lithography steps, these filters do not currently meet industry standards for multispectral filters due to their limited optical properties e.g. low transmission values, or limited spectral range of filters.

The Fabry–Perot (F–P) cavity structure which consists of an intermediate dielectric layer sandwiched by two metallic reflectors, is a simple yet promising candidate in multispectral transmission and reflective filters [16], [17], [18], [19], [20], [21], [22], [23], [24], [25], [26], [27], [28], [29]. Recently, a grayscale stencil lithography process was used to control the thickness of the dielectric layer of the F–P cavity structure by tilting (precessing) a shadow mask array of holes of varying sizes and pitch during the physical vapor deposition process [27]. In order to fabricate transmission color filters, the Ag/SiO_2_/Ag structures are promising due to its polarization-independent transmittance with narrow-band full width half maximum (FWHM) [28]. However, a challenge remains in controlling and varying the thickness of the intermediate dielectric layers to achieve multispectral filters on a single chip. A method to achieve height variation is grayscale lithography where the exposure dose is varied as a function of position to achieve a corresponding thickness variation in photoresist or electron-beam resist after development [28], [29]. Direct electron-beam exposure of F–P structures has also been shown to produce height variation without development [30]. However, these methods are less suited for CMOS manufacturing due to the need for multiple exposures, narrow process windows leading to undesired fluctuations in thickness from batch to batch, and/or the use of unconventional processes. Hence, reliance on binary lithography processes, the cornerstone of CMOS manufacturing is preferred.

Here, we introduce a binary lithography and reflow (BLR) process, where a broad range of thickness of the intermediate dielectric as well steps can be obtained by precise design of patterning and by thermal reflow after a standard binary lithography step. Distinct from a previous study by Wang et al. [31], we avoided the need for pixel-confining structures, yet achieved <2 µm sharp transitions at the pixel border and expanded the applicability to micron-scale lithography processes. Three types of patterns i.e. square holes, lines and meshes were studied, and the minimum feature of the patterns was as large as 600 nm which is comparable to the photolithography pattern in contrast to the previous report where only grating structures with small feature (≤150 nm) were studied. Herein, we use binary lithography to produce regions with patterns of varying density or filling fraction. We have presented a reduced-reflow-process time ≤2 min yet achieved ∼1 nm of surface roughness which provides another advantage in fabrication. A broad range of thickness i.e. 15–200 nm was achieved as well as the step between pixels were more systematically controlled by choosing the patterning structure. Subsequent heating of the sample causes the resist to reflow to form continuous films with thicknesses that increase linearly with the filling fraction. Poly methyl methacrylate (PMMA) was used as the intermediate dielectric layer as proof of concept, due to its behavior as (1) a suitable dielectric material with good transparency, (2) a commonly used electron-beam or photoresist, and (3) a thermoplastic with ability to reflow when heated [32], [33]. Distinct from previous work on grayscale lithography, the intention of this work is to produce regions with flat and uniform thicknesses instead of achieving complex 2.5 D structures. The present BLR method can be useful but not limited to fabricating transmission and reflective spectral filters.

## 2 Materials and methods

### 2.1 Sample fabrication

A 24-nm thick Ag film was sputtered on a glass substrate. A 210 nm-thick PMMA A4 resist with molecular weight of 950,000 was spin-coated on the Ag layer and then baked on the hot plate at 180 °C for 2 min. Three types of patterns having 50 µm sized 16-pixel pitches were written by Raith eLINE Plus electron-beam lithography system. The beam current was 0.534 nA, and acceleration voltage was 30 kV. The exposure dose for the patterns was 400 μC/cm^2^. After the EBL exposure, the patterns were developed by 1:3 methyl isobutyl ketone (MIBK): isopropyl alcohol (IPA) at room temperature for 10 s and then rinsed with IPA for 10 s and finally dried by blowing nitrogen (N_2_) gas. The specimen was then kept on a hot plate to perform reflow process at three different conditions: 180 °C for 30 s, 180 °C for 2 min and 250 °C for 30 s. After the PMMA layer was reflowed, a 24-nm thick Ag layer was sputtered as top mirror to complete the spectral filter.

### 2.2 Charecterization and calculation

The International Commission on Illumination (CIE) standard illuminant D65 was used for the calculation of the CIE chromaticity coordinate from the relevant spectra. The thickness of each pixel of the PMMA film was measured in a microscopic ellipsometer by Accurion. With the help of the region of interest (ROI) tool of the ellipsometer, the measured spot size was kept at 40 μm × 40 µm, and it was aligned to each pixel [34]. Cauchy’s dispersion model was used to fit the refractive index of the PMMA film and thickness was fitted accordingly. Thickness of the Ag film was measured by fitting with Palik’s refractive index data. The reflectance and transmittance spectra of each pixel was measured using a Craic microspectrophotometer. Full-wave electromagnetic simulations were performed using the FDTD simulation software (www.lumerical.com). A plane-wave source incident normal to the sample surface was considered. Spectral transmittance was captured using a power monitor placed below the sample. A Filmetrics Profilm 3D optical profilometer was used to get profilometry data of the pixels.

## 3 Results and discussions

**Figure 1:**
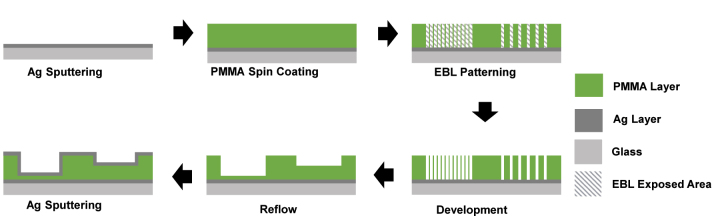
Schematic illustrating the process flow of fabricating transmission color filters.

Figure 1 shows a process flow of the proposed method to fabricate transmission color filters. Details of the fabrication process are in the experimental section. Briefly, a 24-nm thick Ag film is coated on a glass substrate forming the bottom metallic layer of the F–P cavity. A 210 nm-thick positive-tone PMMA film is spin coated as the dielectric layer of the MIM structure. A 3 by 16 array of 50 × 50 μm squares is patterned by electron beam lithography with a chosen infill pattern of varying density. Patterns consist of squares, lines, or meshes. The sample was then developed to form corresponding structures of square holes, trenches or square posts in PMMA. The sample was heated on a hot plate under three different conditions: 180 °C for 30 s, 180 °C for 2 min and 250 °C for 30 s to induce reflow of the patterned PMMA to soften and merge into films of varying thickness depending on the initial filling factors. We later observed that 180 °C for 2 min is close to optimal. In the entire patterning process, the exposure dose was kept constant. Thus, the thickness of the final dielectric film, d, is controlled by the design of the pattern in binary lithography. The Ag film is finally deposited to complete the F–P cavity structure, resulting in closely spaced color filter arrays with varying transmittance spectra (see Figures S1 and S2 for detail).

**Figure 2:**
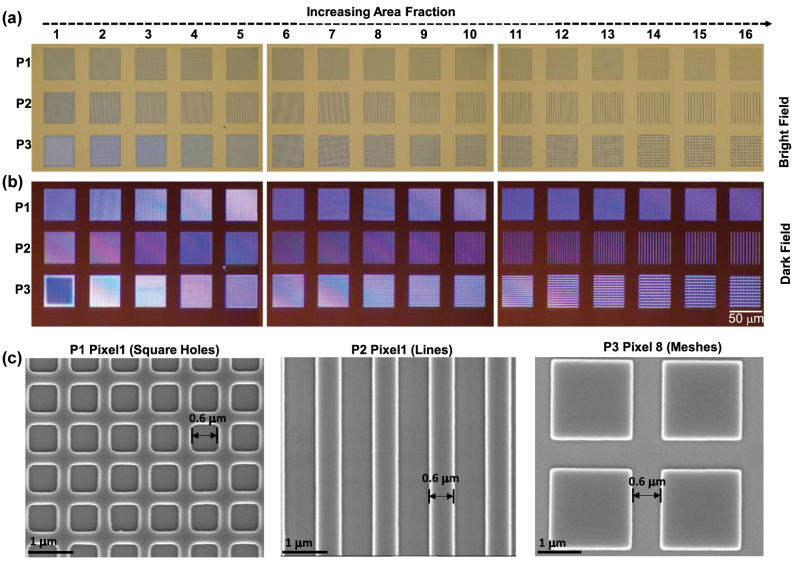
Optical micrographs (OM) and scanning electron microscopy (SEM) images of three types of patterns. The (a) brightfield and (b) darkfield OM images of the 16 pixels of P1 (square holes), P2 (lines) and P3 (meshes). (c) SEM images of P1 pixel 1, P2 pixel 1 and P3 pixel 8.

Figure 2 shows optical microscope (OM) and scanning electron microscope (SEM) images after the three types of patterns were developed. A 210 nm thick PMMA was spin coated as the initial layer, and sub-micron structures were patterned in it. We studied three designs consisting of square holes, lines, and mesh patterns corresponding to pattern-1(P1), pattern-2 (P2) and pattern-3 (P3). Figure 2(a) and (b) are the brightfield and dark field images of these three types of patterns. We considered 16 pixels with varying pitches for all three patterns and the pixel-to-pixel distance was 20 μm for all three designs. In P1, we patterned squares with a constant nominal width, *x* = 0.6 μm, such that pattern density was varied by varying the distance between these squares. With PMMA as a positive resist, these exposed square patterns translate into holes after development. Hence, by volume conservation, as the density of holes increases, the resulting film thickness decreases after the BLR process. Similarly, both P2 and P3 consist of lines also with width *x* = 0.6 μm. In P2, lines were formed in only one direction while in P3, lines in both *x* and *y* directions were patterned, resulting in isolated PMMA islands. Figure 2(c) shows SEM images of three representative pixels for unit cells of P1, P2 and P3.

**Figure 3:**
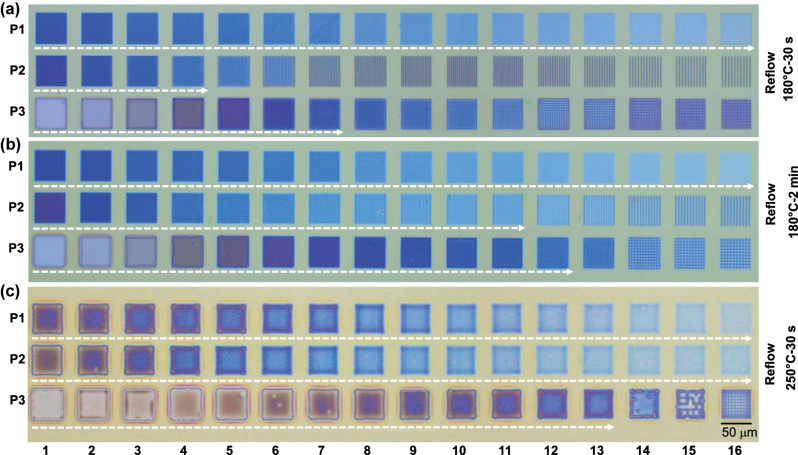
The optical microscopy (OM) images of P1, P2 and P3 printed on Si substrate, after the reflow-process was carried out: (a) reflow at 180 °C – 30 s, (b) reflow at 180 °C – 2 min and, (c) reflow at 250 °C – 2 min, respectively. The white arrows indicate the range of pixels that forms flat surfaces during the reflow process.

The OM image of pixels after the reflow process is shown in Figure 3. Three reflow conditions: 180 °C for 30 s, 180 °C for 2 min and 250 °C for 30 s are presented in Figure 3(a)–(c). We observe different reflow behaviors in P1, P2 and P3. For instance, P1 reflowed earlier than P2 and P3, with all regions fully forming continuous films after 30 s at 180 °C, as indicated by the white arrows (also see Figure S3). The rate of reflow was higher for higher pattern density or lower fill factor (Figure S4). The optical profilometry was conducted and it is found that the reflowed surface is considerably flat with roughness value of ∼1 nm (Figure S5). In contrast, the lower density patterns in P2 required a longer heating duration of 2 min to form a continuous film, with the sparsest patterns never forming films unless heated to 250 °C. P3 exhibited the lowest tendency to form continuous films. However, as it had the lowest filling fraction of PMMA patterns, it achieves the thinnest films of the three patterns. The films increase in thickness from left to right. At 250 °C – 30 s, all the pixels are formed, due to the fluid nature of PMMA at this temperature but exhibit a thickness gradient at the interpixel boundary. It is noticed that the intensity of the reflow process is higher at the center comparing to the edge (Figure S4). Therefore, at 250 °C, which is an over-reflowed state, the material accumulation at the center continues thus producing a bowing like shape at the center. We noticed that the reflow process works for a limited set of patterns, and is strongly dependent on the pitch of the patterns. For patterns with pitch >3 μm, reflow was limited and pixels could not reflow into flat surfaces at 180 °C – 2 min. Thus, if the lines (P2) are far from each other, it requires higher energies to reflow compared to the closed placed lines. No shrinkage or dewetting of the film is noticed during the reflow process.

The thicknesses of all the pixels were measured by ellipsometry and plotted against the filling fraction of all patterns with comparison with a grayscale approach, which shows a strongly nonlinear behavior (Figure S6). The nominal area fractions of the square and meshed patterns (P1 and P3) and actual area fraction of lines (P2) exhibit a linear relation with the final thickness after the reflow process. The area fraction of the patterns can be related to the fill factor of patterns. As the pitch of the patterns decreases for a fixed *x*, the area fraction of PMMA decreases. The thickness of each pattern can be controlled with a linear relation to the areal filling fraction between ∼15 and 200 nm, corresponding to ∼8–95 % of the original resist thickness. The proposed BLR method exhibits more scope in terms of thickness variation due to its linear relation to area fraction as well as more controllability in terms of thickness step. In contrast, exponential decrease of PMMA thickness with varying dose is noticed as well as controllability is less in terms of thickness step in case of grayscale.

**Figure 4:**
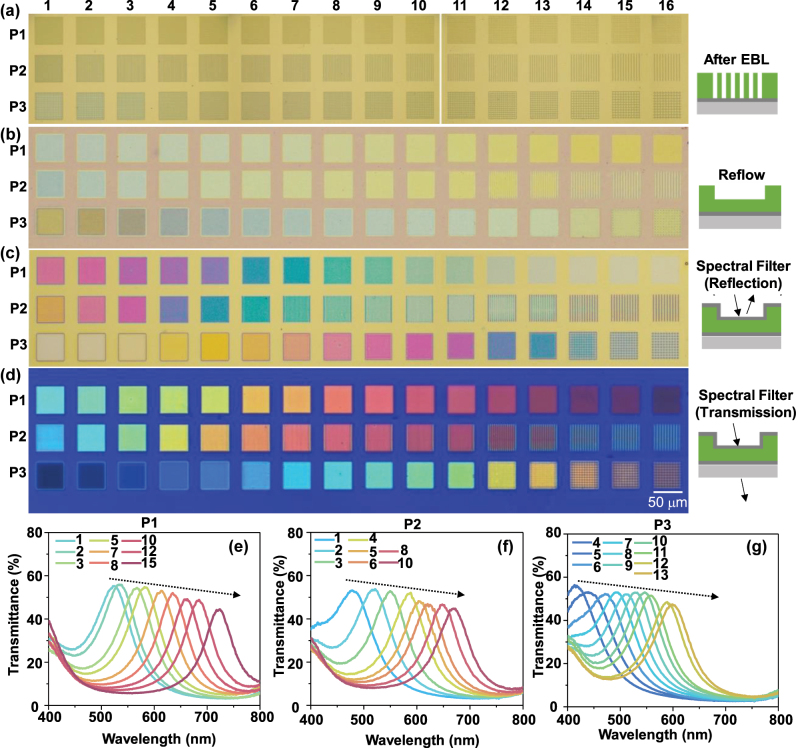
Brightfield OM of the three patterns in each step of spectral filter fabrication and corresponding transmission spectra. (a) OM of the pattern-1, pattern-2, and pattern-3 printed on the PMMA/Ag/Glass structure, images are taken after the patterns are developed. (b) Brightfield OM of the same sample after the reflow process, and (c) after Ag coating on the reflowed PMMA/Ag/Glass structure. (d) Transmission OM of the fabricated filters by Ag/PMMA/Ag/Glass. The measured transmission spectra of the filters fabricated by (e) P1, (f) P2, (g) P3. The arrow in (e), (f) and (g) indicate the trend of the spectra with variation of nos. of pixels in three patterns.

Figure 4 shows brightfield OM images of the filters at various stages, along with transmission color images, and measured transmission spectra. Figure 4(a) presents OM images of P1, P2 and P3 printed on PMMA (210 nm)/Ag/Glass. Figure 4(b) exhibits the optical micrographs after the reflow process of the same structures shown in Figure 4(a). Figure 4(c) shows OM images after the Ag top layer is coated. The series of colors generated for each pixel provides visual evidence of the systematic step creation of PMMA by reflow process. The transmission color images are shown in Figure 4(d). The transmission spectra of the highlighting color pixels span the visible spectrum from 450 to 750 nm wavelengths at ∼50 nm steps as shown in Figure 4(e)–(g). The measured transmission spectra of F–P cavity structure formed by these patterns show similar transmittance bands as simulated and presented in Figure S1. Figure S7 represents the transmission and the reflection colors in the CIE 1931 chromaticity coordinates for the fabricated color pixels of three patterns. The absorptance spectra of pixel1 of P2 was calculated by subtracting the measured reflectance and transmittance spectra from incident light intensity agree well with simulated spectra for Ag (24 nm)/PMMA (109 nm)/Ag (24 nm) structures (Figure S8). Meanwhile, the study introduces a novel method for controlling PMMA resist's step height through binary lithography and reflow (BLR). Unlike the other conventional approaches such as grayscale, which lacks repeatability and is impractical for industrial applications due to its sensitive dose requirement, BLR offers a one-time exposure and reflow process to achieve step heights, reducing fabrication steps and increasing process latitude for industry application.

## 4 Conclusions

The BLR process presented here enables a single lithographic step to control the thickness of F–P cavities, suited for scalable manufacturing of multiple high-efficiency transmission color filters. The Ag/PMMA/Ag structure was considered as F–P cavity structure and the optical filters span visible spectrum. Three designs were considered for the patterning on the PMMA/Ag, followed by reflow process to provide systematically controlled cavity steps. As the exposure dose of EBL was constant, the pattern-design and fill factor determine the thickness of the cavity. Remarkably, the resulting thickness spanning ∼8–95 % of the original PMMA thickness could be achieved. Accurate thickness control with increments of ∼6 nm was achieved by varying the pitch of the binary pattern. Though we have shown simple rectilinear patterns exposed using EBL in PMMA, this work can be extended to more sophisticated patterns for better reflow behavior, and patterning using other lithographic methods, e.g. projection lithography or nanoimprint lithography, with other suitable polymers or resins.

## Supplementary Material

Supplementary Material Details
