# Supplementary material for: Scaling up multispectral color filters with binary lithography and reflow (BLR)
Source: Nanophotonics. 2024 Jun 3;13(19):3671–7. doi: 10.1515/nanoph-2024-0090 (PMC11465983; doi:10.1515/nanoph-2024-0090)
Supplement: Supplementary file 1 — Supplementary Material Details [file j_nanoph-2024-0090_suppl_001.docx]

Supplementary information

Scaling Up Multispectral Color Filters with Binary Lithography and Reflow (BLR)

*Md Abdur Rahman^a^, Soroosh Daqiqeh Rezaei^a^, Deepshikha Arora^a^, Hao Wang^a^, Tomohiro Mori^b^, Ser Chern Chia^a^, John You En Chan^a^, Parvathi Nair Suseela Nair^c^, Siam Uddin^a^, Cheng-Feng Pan^a^, Wang Zhang^a^, Hongtao Wang^a^, Zheng Ruitao^d^, Lim Sin Heng^d^, and Joel K.W. Yang^a^**

^a^ Engineering Product Development Pillar, Singapore University of Technology and Design, 8 Somapah Road, Singapore 487372, Singapore.

^b^ Industrial Technology Center of Wakayama Prefecture, Wakayama 6496261, Japan.

^c^ Institute of Materials Research and Engineering, A*STAR (Agency for Science, Technology and Research), 2 Fusionopolis Way, #08-03 Innovis, Singapore 138634, Singapore.

^d^ Lite-On Singapore Pte. Ltd., New Tech Park, Singapore 556741, Singapore.

*Email: joel_yang@sutd.edu.sg

THEORETICAL ANALYSIS

Full electromagnetic wave simulation of the transmission spectra of an Ag(24 nm)/PMMA(*d*)/Ag(24 nm) was performed using finite-difference time-domain (FDTD) simulation. Figure S1(a) presents the schematic of the Ag(*m*)/PMMA(*d*)/Ag(*m*) structure used to simulate transmission spectra. We used glass (SiO_2_) as the substrate, though an actual implementation of this method would require patterning directly onto CMOS photodetectors. Simulation was carried out using Palik’s refractive indices of Ag thin film [1]. The simulated transmission spectra of Ag/PMMA(*d*)/Ag structure on glass with varying *d* and *λ* are presented in Figure S1(b). Herein, as *d* is varied from 92 to 228 nm with 17 nm increments, the corresponding narrow band transmission with a transmission peak from 450 nm to 850 nm at intervals of 50 nm is obtained. In order to decide the thickness of Ag layer that required for the fabrication of the transmission color filter, the dependence of the transmission spectra on the wavelength was also simulated for the Ag(*m*)/PMMA(109 nm)/Ag(*m*) structure with varying *m* from 18 nm to 28 nm and at intervals of 2 nm and presented in Figure S1(c). The FWHM and transmission peak values for the spectra shown in Figure S1(c) are presented in Figure S1(d). As *m* increases from 18 to 28 nm, both FWHM and transmission peak values decrease from 172 to 92 nm and 72% to 56%, respectively. Therefore, the 24-nm thick Ag film was found to be more suitable as a metal reflector for the spectral filter fabrication while taking both FWHM (117 nm) and transmission peak value (64%) into consideration. As *m* increases, the wavelength corresponds to transmission peak also blueshifts, 515 nm to 492 nm for increasing *m* from 18 to 28 nm, as presented in Figure S1(e). As *d* increases for a fixed *m*, the transmission peak redshifts in order to fulfill the resonance condition in the F-P cavity (Figure S1(f)) [2]. The dependency of the transmission spectra with varying incident angle (θ) from 0 to 25 degrees for the Ag(24 nm)/PMMA(109 nm)/Ag(24 nm)/Glass structure is presented in Figure S1(g). We observe that the transmission spectra exhibit a blueshift of ~1 nm per degree tilt. Generally, interference filters will blue-shift due to an effective phase-delay reduction in the dielectric layer for larger angles [3]. Simulation results presented here are in agreement with similar structures in literature for varying incident angles [4–6]. Colors in transmission were calculated from the spectra shown in Fig S1(b) and presented in the CIE 1931 chromaticity as shown in Figure S2.


**Figure S1.** (a) Schematic of the Ag(*m*)/PMMA(*d*)/Ag(*m*) structure. (b) The transmission spectra of Ag/PMMA(*d*)/Ag structure on glass with varying *d* and *λ*. (c) The transmission spectra of Ag(*m*)/PMMA(109 nm)/Ag(*m*) structure on glass with varying *m* and *λ*. (d) The FWHM and transmission peak values for the Ag(*m*)/PMMA(109 nm)/Ag(*m*) structure on glass with varying *m.* (e) The transmission peak wavelength for the Ag(*m*)/PMMA(109 nm)/Ag(*m*) structure on glass with varying *m.* (f) The transmission peak wavelength for the Ag (*m*)/PMMA (109 nm)/Ag(*m*) structure on glass with varying *d.* (g) The simulated transmission spectra of Ag(24 nm)/PMMA(109 nm)/Ag(24 nm)/Glass structure varying incident angle (*θ*) from 0 to 25 degrees. In Figure S1, the terms Sub. and Trans. represent substrate and Transmittance, respectively.

**Figure S2.** The simulated transmission colors in the CIE 1931 chromaticity coordinates for the Ag(24 nm)/PMMA(*d*)/Ag(24 nm) structure on glass with increasing *d* as indicated by the arrow.

**Figure S3.** The SEM image of P1 pixel 4 after the reflow process was completed at 180°C - 30 s. (a) the overall image of the pixel. (b) zoomed image of the marked corner of the pixel. It is noticed that all the islands were melted after the reflow.

**Figure S4.** The SEM image of (a) P1 pixel 14 and (b) P1 pixel 16 after the reflow process was conducted at 180°C - 30 s which suggests that the reflow process yet not completed.

**Figure S5.** The optical profilometry data of P1 pixel 5 after the reflow process was conducted at 180°C - 30 s: (a) line profile data where inset showing optical image of the pixel, (b) top view (2D) and side view of the pixel. Here, yellow colored mark on top view image shows where area roughness was measured.

**Figure S6.** (a) The thickness of the pixels with varying nominal area fraction of PMMA of square (P1) and mesh structures (P3). (b) The thickness of the pixels with varying area fraction of patterned PMMA with grating structures obtained from OM image. (c) The grayscale test with EBL: PMMA thickness vs exposure dose variation.

**Figure S7.** The measured transmission (top row) and reflection (bottom row) colors in the CIE 1931 chromaticity coordinates for the fabricated color pixels of three patterns. (a), (b) and (c) are the transmission colors and (d), (e) and (f) are the reflection colors for P1, P2 and P3, respectively.

**Figure S8.** The absorbance spectra of the pixel 1 of P2 compared with spectra calculated for Ag(24 nm)/PMMA(109 nm)/Ag(24 nm) structure. Here, the normalized absorbance was extracted from the reflectance and transmittance data of the corresponding pixel considering A=1-T-R.

REFERENCES

(1) Palik, E. Handbook of Optical Constants of Solids (New York: Academic Press) **1997**.

(2) Rahman, M.; Kim, Y. H.; Cho, S.; Lee, S.; Byun, J. Realization of Structural Colors via Capped Cu-based F−P Cavity Structure, *Opt. Express* **2021**, 29, 29466–29480.

(3) Frey, L.; Masarotto, L.; Armand, M.; Charles, M.; Lartigue, O. Multispectral interference filter arrays with compensation of angular dependence or extended spectral range. *Opt. Express*, **2015**, *23*, 11799 –11812.

(4) Lin, Y. C.; Chen, Z. A.; Shen, C. H. The Simulation and Fabrication of Ag/SiO_2_/Ag Thin Films Color Filter. *Phys. Procedia*, **2012**, *32*, 19–30.

(5) Wang, Y.; Zheng, M.; Ruan, Q.; Zhou, Y.; Chen, Y.; Dai, P.; Yang, Z., Lin, Z.; Long, Y. Li, Y.; Liu, N.; Qiu, C.; Yang, J.; Duan, H. Stepwise-Nanocavity-Assisted Transmissive Color Filter Array Microprints. *Research* **2018**, *2018,* *8109054(1)* –*8109054(10)*.

(6) Williams, C.; Gordon, G.; Wilkinson, T.; Bohndiek, S. Grayscale-to-Color: Scalable Fabrication of Custom Multispectral Filter Arrays. ACS Photonics **2019**, *6*, 3132–3141.
